# 2280: Preliminary evaluation of postural stability as a cost-effective means of quantitatively and objectively differentiating between autism spectrum disorder, developmental coordination disorder, and typical development

**DOI:** 10.1017/cts.2017.102

**Published:** 2018-05-10

**Authors:** Haylie Miller, Nicoleta Bugnariu, Priscila Caçola, Rita Patterson

## Abstract

OBJECTIVES/SPECIFIC AIMS: Individuals with autism spectrum disorder (ASD) and developmental coordination disorder (DCD) share overlap in their motor symptom profile and underlying neurology (Sumner, Leonard, & Hill, 2016, JADD). DSM-5 guidelines allow these 2 disorders to be independent or co-occurring (APA, 2013), but common clinical practice does not include systematic assessment to determine the presence or absence of co-occurring DCD in children with ASD, or vice versa. Furthermore, in many cases DCD is managed in a nonspecific manner, with schools making accommodations for a child’s motor challenges without formally assigning a diagnosis of DCD. Thus, somewhat subjective, qualitative judgments are made by clinicians to classify children as DCD, ASD, or ASD+DCD in the absence of a reliable, valid, quantitative measure to distinguish between these profiles. As a first step toward developing such a measure, researchers must tease apart the nuanced differences in the motor symptoms of these 2 developmental disorders using methods that are scalable to clinical and educational settings. These methods must also be developed with consideration for logistical variables such as cost, clinical utility of data output, and ease-of-use if they are to be transferrable to physicians, school nurses, and other community health workers outside of academic laboratory settings. To that end, we conducted 2 complementary studies: 1 in the lab and 1 in the community. METHODS/STUDY POPULATION: In the community-based study, we used an affordable, user-friendly, portable balance testing system to assess postural stability during quiet standing (feet shoulder-width apart) with eyes open for 30 seconds. Data were generated from a single force plate in the balance platform. Potential participants were screened for other medical and neurological conditions that might impact their postural stability, and those with significant comorbidities were excluded. We tested 15 children with a reported diagnosis of ASD, 8 children with suspected or diagnosed DCD who were enrolled in a motor intervention program, and 30 typically-developing (TD) children with no significant developmental history reported. The ASD group ranged in age from 7 to 20, the DCD group ranged from 7 to 10, and the TD group ranged from 7 to 19. In the lab-based study, we again obtained force plate data during quiet standing (feet shoulder-width apart) with eyes open for 30 seconds, in our system that also included full-body motion capture, virtual reality, and mobile eye tracking. (Data from these additional sources are not discussed in this disseminaton, as our current focus is on identifying a simple, scalable metric that can be used to distinguish ASD from DCD.) We tested 10 children with a diagnosis of ASD that was confirmed by the research team, 10 children with a diagnosis of DCD that was confirmed by the research team, and 5 TD children with no significant developmental history reported. The ASD group ranged in age from 7 to 18, the DCD group ranged from 8 to 12, and the TD group ranged from 9 to 18. RESULTS/ANTICIPATED RESULTS: Primary outcome measures in both studies were related to Center of Pressure (CoP), including CoP sway, CoP velocity, and amount of sway relative to the base of support. Data analysis from both studies is ongoing, but preliminary trends suggest that CoP metrics may effectively differentiate between ASD, DCD, and TD. During quiet standing, individuals with DCD exhibited the greatest postural sway. Individuals with ASD followed, having greater instability than the TD group. Differences were also evident in the velocity profiles of postural sway. DISCUSSION/SIGNIFICANCE OF IMPACT: Preliminary findings suggest that CoP offers a means of differentiating between typical and atypical development specifically with respect to motor symptoms. This simple, quantifiable measure may prove a sensitive and specific means of systematically assessing co-occurrence of ASD and DCD in clinical and educational settings, leading to more accurate diagnostic classification and tailored intervention. Future directions include conducting analyses that account for participant age and developmental stage with respect to motor skills, determining whether trends hold in a larger sample, and using advanced statistical methods to determine whether CoP variables have predictive validity in discriminating between classifications of ASD, DCD, ASD+DCD, and TD. Eye-movement data were also obtained during these tasks, and may further aid in understanding the factors contributing to atypical postural control. These 2 studies also yielded methodological findings, namely that the portable force platform carries the benefit of high ease-of-use, low cost, and portability, but also has important drawbacks. Specifically, it is not capable of registering accurate CoP data for participants who weigh <40 lbs, and the error variance for the load cells is greater than that of most nonportable, higher-end plates like those embedded in our laboratory’s platform. As technological advances continue to facilitate development of more portable, higher-resolution systems, these drawbacks may be significantly reduced. Future directions include further assessment of portable, affordable solutions for this type of testing to identify whether higher-resolution options are available, whether this added resolution increases classification accuracy, and how ease-of-use is perceived by clinical and community health workers.

